# The Treatment of Sensitive Dentin, with Special Reference to the Production of Anesthesia by Pressure

**Published:** 1907-04-15

**Authors:** W. D. Miller

**Affiliations:** Berlin, Germany


### THE TREATMENT OF SENSITIVE DENTIN, WITH SPECIAL REFERENCE TO THE PRODUCTION OF ANESTHESIA BY PRESSURE.

BY W. D. MILLER, M. D., D.D.S., BERLIN, GERMANY.

There is one point in the treatment of sensitive dentin which may be considered as definitely established, namely, that by the use of sharp instruments, cutting with the least possible amount of pressure and consequent development of heat, and by thoroughly drying the tissue to be operated upon with warm air in connection with absolute alcohol, we are able to greatly reduce the sensitiveness of the dentin in the majority of cases which come under our treatment.

Other physical agents for obtunding the dentin, such as heat, cold, electricity, have been and are still used occasionally, with varying degrees of success, and a great number of chemical agents have been recommended as adjuncts to dryness and sharp instruments. Of these I will only say that a solution of equal parts of chloroform and crystallized chlorid of zinc, with an addition of cocain *ad libitum*, has given me the best results in cavities of decay. For superficial sensitiveness at the necks of the teeth, nitrate of silver, applied in substance or concentrated solutions and the part protected afterward for a few hours by a covering of Fletcher's artificial dentin, works admirably. Dr Abbot assures me that the chlorid of zinc applied in the same way gives especially good results without the discoloration.

In Germany considerable use has recently been made of various preparations of the suprarenal capsule in combination with cocain, injected subgingivally in the treatment of sensitive dentin, and the German dental journals of 1904, 1905 are full of articles on suprarenin-cocain, adrenalin-cocain, eusemin, etc. The suprarenin-cocain has been prepared after the formula of Braun in tablets containing 1.0 centrigram cocain and 0.1 milligram suprarenin. A tablet dissolved in ½ ccm. of boiled water injected in the neighborhood of the apex of the tooth will, in a great majority of cases, especially in the upper jaw, produce complete anesthesia of the dentin. These preparations are, however, not without their disadvantages; occasionally cases of poisoning have been observed or of sloughing of the gums, and it is claimed by some that the excessive anemia of the pulp eventually results in its death.

More recently novocain has been highly recommended instead of cocain. It is claimed that it is far less poisonous than cocain and nearly equal to it in its anesthetic action. Misch recommends the following combination:

| | | |
|---|---|---|
| | Novocain | 0.1 |
| | Physiological salt solution | 10.0 |
| | Suprarenin boric (1.4000) | 10 drops |
| or | Novocain | 0.1 |
| | Physiological salt solution | 5.0 |
| | Suprarenin boric (1.4000) | 5 drops |

But even the novocain-suprarenin combination has its dark side. Hamecher (*Odontologische Blatter*, July, 1906) writes that the pain following the injection is often so severe that the patients affirm that they can not bear it. By an injection of Stovain the pain ceases almost immediately, to return later with increased intensity. Two tablets of aspirin of ½ gram (7½ grains) each, relieve the pain permanently. He observed repeatedly considerable swelling; the edema is painful and lasts as long as eight days. Paralysis of the facial nerve was observed four times; it disappeared in a few hours. General complications not infrequently present themselves, such as a feeling of fever, beating of the heart, dizziness, asphyxia, etc. These symptoms disappear only gradually, and often headache remains till the following day. The ill effects are exaggerated, but still it appears that the injection of novocain-suprarenin and similar combinations *for anesthetizing the dentin* is not so perfect in its results as to obviate the desirability or necessity of improving our more simple methods and applications.

It has always appeared to me that our knowledge of the means of obtunding sensitive dentin is for the most part empirical. Almost every known anesthetic has been tested practically in the mouth and naturally with conflicting results, because the conditions are so diverse in different cases and so many concomitant factors, such as the degree of dryness, sharpness of the instruments, skill of the operator and the hypnotic effect on the patient cannot be eliminated. Consequently, notwithstanding the millions of experiments that have actually been made in the mouth, we do not yet know what is the best local application for obtunding sensitive dentin.

It seems to me that we ought to be able to accomplish something by experiments on freshly extracted teeth, and in this hope the work to be described was undertaken. The action of chemical agents upon sensitive dentin will depend in a great measure upon the depth to which they penetrate, and it becomes a very important matter to determine. *Under what conditions do we obtain the deepest penetration?* In the first place , the attempt was made to determine the effect of dryness on the penetration. In order to obtain perfectly homogeneous material, blocks of ivory were superficially decalcified and some of them thoroughly dehydrated by use of absolute alcohol and then immersed in a solution of eosin along with others not dehydrated. I was much astonished to find that almost no penetration whatever took place in the former, which I account for by the fact that the alcohol caused intense shrinkage of the decalcified tissue and partial obliteration of the tubules. Drying with chlorid of lime gave better results. I did not feel at liberty, however, to conclude from these results that the use of alcohol produced the same effect on human teeth, inasmuch as in caries of the dentin changes take place which make it differ materially from simply decalcified ivory. The experiments were accordingly continued on freshly extracted human teeth. A large cavity of decay was excavated partially and a thin wall of wax built across from one side to the other (not a simple matter, by the way). One of the two partitions thus formed was dehydrated by alcohol, the other left moist. Eosin or picrofuchsin was now flowed into the separate partitions and after a lapse of 2 to 18 minute sections made at right angle to the partitions to determine on which side the penetration had been deeper. As this procedure turned out to be a very difficult one, I finally adopted the plan of simply sawing the tooth into halves and treating each half separately. As a result of a great number of experiments, I found that *as far as the penetration* is concerned it made very little difference whether I applied the solution to a moist cavity or to one previously dried with alcohol. Sometimes a slight advantage appeared to be on one side and sometimes on the other. *In practice we would obtain better results by operating on the dry dentin, since the drying of itself destroys the conductivity of the dentinal fibrils.*

On a second series of experiments the comparative penetration ot different solutions in water, alcohol, glycerin, peroxid ot hydrogen, formalin, chlorid of zinc. etc., was tested on freshly extracted human teeth. Here I found such slight differences that I came to the conclusion it matters but little in which vehicle we dissolve our anesthetic. In some preparations there seemed to be a slight advantage in favor of alcohol, while in others the aqueous solution penetrated more deeply. In particular, my hopes that a solution in peroxid of hydrogen would permeate the dentin very rapidly were not realized. Between glycerin and water I found a slight advantage in favor of the water. Glycrine has been recommended as a vehicle for anesthetics, but in my experiments I found almost invariably the aqueous solution penetrated a little deeper than glycerin, and consequently consider an aqueous solution preferable.

The following idea appears to me to merit consideration in this connection: If we wish to fill any porous compressible body with a liquid, we first exert pressure upon it, by which we empty its pores, and by then immersing it in the liquid it will, by virtue of its elasticity, resume its original shape and fill itself with the liquid. It occurred to me that we might apply this principle to our operations by flooding the cavity with the solution and then exerting interrupted pressure upon the carious dentin. I found by experiments that by going over one-half of a cavity with a burnisher, exerting pressure on the dentin, as if packing amalgam, I could obtain a greater depth of penetration than in the half where this manipulation had not taken place. I have not tried this in practice, though I believe it has been recommended in the dental journals. It is possible that if too great pressure were exerted pain would be produced, but I have no doubt that by this method the penetration of the anesthetic would be increased just as well in the mouth as out of it.

One of the methods of procedure which has caused the greatest amount of discussion in the last two or three years is injection into or through the dentin by means of high pressure, obtained by one of the various syringes that have been introduced for this purpose. It is claimed that by instruments such as the Wilcox-Jewett syringe one can produce a pressure of 3,000 pounds to the square inch. It is claimed that one can obtain perfect anesthesia of the pulp inside of a minute or two, and that is quite true.

I heard from a colleague that many cases had been observed where inflammation of the pulp followed the application of this instrument. The thought suggested itself to me that it might not be necessary to use a pressure anything like 3,000 pounds to the square inch. A pressure of 500 pounds might not even be necessary. I found that by taking a rubber tube, drawing the end over the crown of a tooth and ligating it and putting a few drops of an aqueous solution of eosin in the rubber tube, and simply pressing the tube with the fingers, I could press the solution through the dentin and thus stain the pulp. It is plain, therefore, that very little pressure is necessary to force anesthetic solutions through the dentin and bring about a more or less complete anesthesia.

When it comes to applying this principle in practice a great difficulty arises. As you all know, various attempts have been made to anesthetize not only the pulp, but the dentin, by pressure, which is produced by the simple method of putting a pledget of cotton saturated with the solution into the cavity, covering it with unvulcanized rubber and pressing upon it with a burnisher. The great difficulty is that the solution oozes out around the margins of the cavity, leaving the cotton dry under the burnisher. Very often we obtain less penetration in that way than if no pressure is used at all. In a letter I received from Dr. Jenkins, he told me he has obtained good results by first introducing his anesthetic on cotton, covering this with unvulcanized rubber, and then drawing a polishing strip across the tooth, forcing the rubber against the margins of the cavity so as to obtain a fairly good contact, and then applying pressure by a flat burnisher. By that means he has been able to anesthetize the dentin.

For a long time I have been in search of a method by which we could keep a solution in a cavity of decay and exert a moderate pressure upon it. We do not need two or or three hundred diameters. Half an atmosphere, the pressure of the finger, will suffice. I thought of soft rubber cup-shaped points of different shapes, approximately the size of the cavity, with holes bored through them, into which we could insert the point of a minature syringe. Finally the thought struck me that we might accomplish the purpose in the following way. After excavating the cavity as far as convenient and smoothing the borders a bit, take an impression with Stents' composition, endeavoring to get the margin of the cavity fairly well brought out; put a few threads of cotton into the cavity and saturate them thoroughly with a 5 to 10 per cent solution of cocain, cover this with a small bit of rubber dam, and then press the Stents' impression down upon it. We obtain thereby a perfect closure of the margin, so that the liquid cannot escape, and one can then exert pressure with the thumb sufficient to press the solution into the dentin. I went immediately into my laboratory and tried the experiment on freshly extracted teeth and found I could press the solution through two millimeters, while breathing twenty times (about 1½ minutes) in a sufficient quantity to stain the pulp superficially. I got hold of this idea only two days ago, so that I have not had much opportunity of trying it in practice. I had three cases yesterday, one a bad case of caries of the left lower first molar a sort of leathery decay causing much pain. I attempted, first, by means of a spoon-shaped excavator, to lift out a great flake of softened dentin, but the pain caused was so great that I was obliged to desist. I took an impression and proceeded in the way I have described, and inside of two minutes the anesthesia was so complete that I could excavate that tooth completely without causing the boy any pain. The second case was that of an exposure of pulp in a left upper lateral incisor. I took the impression and proceeded in the same way and was able immediately to extirpate the pulp. The third case was a lower second molar with a cup shaped cavity; the walls were broken down and there was a certain amount of spontaneous healing of the dentin. In those cases it is difficult to penetrate the dentin. I took an impression and applied the method, but did not succeed in producing complete anesthesia, for the reason just stated. I likewise found that thick layers of softened dentin are penetrated slowly. In a cavity containing a lump of softened leathery dentin, you may apply the anesthetic and wait as long as you please; it will often have little or no effect; especially is this the case where the cavity is greasy. Also, secondary, and to a certain extent transparent, dentin are permeated with great difficulty. Secondary dentin forms almost an absolute barrier to penetration.

The point I wish particularly to make is that it has been demonstrated with sufficient certainty that we may, by use of *very little* pressure, force solutions through the dentin, and I hope that others of greater practical ingenuity than I I possess will be able to devise methods of applying such pressure in the different classes of cavities which we have to deal with in practice.—*Dental Review.*

